# Associations between access to recreational physical activity facilities and body mass index in Scottish adults

**DOI:** 10.1186/s12889-016-3444-8

**Published:** 2016-08-09

**Authors:** Anne Ellaway, Karen E. Lamb, Neil S. Ferguson, David Ogilvie

**Affiliations:** 1 MRC/CCSO Social & Public Health Sciences Unit, University of Glasgow, 200 Renfield Street, G2 3PG Glasgow, UK; 2 Deakin University, Melbourne, Australia; 3 University of Strathclyde, Glasgow, UK; 4 MRC Epidemiology Unit and UKCRC Centre for Diet and Activity Research (CEDAR), University of Cambridge, Cambridge, UK

**Keywords:** Obesity, Physical activity facilities, Car ownership, Public transport, Neighbourhood, Neighborhood, Transport, Accessibility

## Abstract

**Background:**

The aim of this country-wide study was to link individual health and behavioural data with area-level spatial data to examine whether the body mass index (BMI) of adults was associated with access to recreational physical activity (PA) facilities by different modes of transport (bus, car, walking, cycling) and the extent to which any associations were mediated by PA participation.

**Methods:**

Data on individual objectively-measured BMI, PA (number of days of (a) ≥20 min of moderate-to-vigorous PA, and (b) ≥15 min of sport or exercise, in previous 4 weeks), and socio-demographic characteristics were obtained from a nationally representative sample of 6365 adults. The number of accessible PA facilities per 1,000 individuals in each small area (data zones) was obtained by mapping a representative list of all fixed PA facilities throughout mainland Scotland. A novel transport network was developed for the whole country, and routes on foot, by bike, by car and by bus from the weighted population centroid of each data zone to each facility were calculated. Separate multilevel models were fitted to examine associations between BMI and each of the 24 measures of accessibility of PA facilities and BMI, adjusting for age, gender, longstanding illness, car availability, social class, dietary quality and urban/rural classification.

**Results:**

We found associations (*p <* 0.05) between BMI and 7 of the 24 accessibility measures, with mean BMI decreasing with increasing accessibility of facilities—for example, an estimated decrease of 0.015 BMI units per additional facility within a 20-min walk (*p =* 0.02). None of these accessibility measures were found to be associated with PA participation.

**Conclusions:**

Our national study has shown that some measures of the accessibility of PA facilities by different modes of transport (particularly by walking and cycling) were associated with BMI; but PA participation, as measured here, did not appear to play a part in this relationship. Understanding the multi-factorial environmental influences upon obesity is key to developing effective interventions to reduce it.

## Background

The prevalence of obesity, which is high in developed countries and rising worldwide, is associated with a significant burden of ill-health including cardiovascular disease, certain cancers and diabetes [1–5], particularly in lower socio-economic groups [6]. Efforts to address key determinants of obesity, such as initiatives to promote physical activity directed at individuals, have had limited success to date [7, 8]. This has led to increasing attention on other potential determinants not specific to the individual [9], including efforts to understand environmental influences, particularly those related to the built environment [10, 11]. However, most studies to date have tended to use relatively simple metrics of accessibility such as proximity to the nearest facility or the density of facilities in the neighbourhood of residence. Few studies have taken account of the fact that individuals may travel further afield to reach facilities, or that access of this kind may be inequitably patterned in that low income groups may be less likely to have a private motor vehicle available to take them to more distant destinations. In addition, few studies have focused on the specific roles of different forms of transport such as cars and public transport on access to physical activity opportunities, which may in turn promote participation and affect subsequent obesity levels [12]. This is despite a longstanding literature on the importance of the availability of public transport for access to health care [13, 14].

Some studies, mainly from the USA and Australia, have shown that distance to physical activity opportunities and perceived availability of transport options appears to influence use [15–17]. Moreover, residents of rural areas may be particularly constrained in their access to a range of goods and services if public transport is limited [18]. In our Scotland-wide study [19] we found that those without car access but living in more affluent neighbourhoods [20] or rural areas where public transport provision is low [21] might be particularly vulnerable to having difficulties in accessing health promoting facilities.

In this paper, we extend our previous work by linking individual health and behavioural data with area-level data on accessibility to examine whether access to recreational physical activity facilities by different modes of transport was associated with the body mass index of adults in Scotland, and the extent to which any association was mediated by physical activity participation.

## Methods

### Study sample and individual-level data

Data on body mass index (BMI), age, gender, social class, car availability, presence of longstanding illness (categorised as limiting longstanding illness, non-limiting longstanding illness or no longstanding illness), physical activity and diet were obtained from the 2003 Scottish Health Survey [22], a survey designed to provide a nationally representative sample of the population living in private households in Scotland. BMI was calculated from measurements of height and weight recorded during a nurse visit as part of the survey. Social class was ascertained using the individual National Statistics Socio-economic Classification (NS-SEC) [23] and categorised as managerial and professional occupations, intermediate occupations, routine and manual occupations or other.

Two potential mediating variables relating to physical activity participation were extracted from the Scottish Health Survey. The first was the number of days in the last four weeks on which at least 20 min of moderate-to-vigorous physical activity (including housework, gardening, do-it-yourself (DIY) and walking) were undertaken. The second was the number of occasions in the last four weeks on which at least 15 min of sport or exercise activity were undertaken. The first variable displayed a bimodal distribution, with many individuals reporting physical activity either every day or not at all during the four-week period, whilst the second was highly skewed with most individuals reporting no occasions of sporting activity. Both variables were therefore grouped into three categories: none, 1–11, and 12 or more (which corresponds approximately to three times per week) for analysis.

The Dietary Quality Index (DQI) was used to adjust for dietary behaviour [24]. The DQI for adults is computed from the self-reported intake of fish, red meat and meat products, starchy foods, fibre, sugary foods, fatty foods, alcohol, and fruit and vegetables. The food groups included in the DQI are based on information from the Scottish Dietary Targets and the guidelines on food intake proposed by the Food Standards Agency, the Scientific Advisory Committee on Nutrition, the World Cancer Research Fund and the World Health Organization. The DQI was grouped into quintiles from 1 (the worst diet) to 5 (the best diet).

The analysis was designed to examine associations between the accessibility of physical activity facilities and BMI in adults. In total, 8,148 (71.0 %) participants in the Scottish Health Survey were aged 16 years or over and thus eligible for inclusion in the analysis. 1,468 (18.0 %) of these adults had missing BMI information or implausible BMI values—the latter defined for this purpose as less than 15 kg/m^2^ (*n =* 6) or greater than 50 kg/m^2^ (*n =* 14)—and were excluded from analysis, as were those with missing values for any of the explanatory variables and mediators. These exclusions resulted in a final sample for complete case analysis of 6,365 individuals, nested within 2,763 data zones (see below) within 29 local authorities. The analysis sample was comparable to the full sample of 8,148 in terms of their socio-demographic characteristics and self-reported physical activity variables (data not shown).

### Area-level and transport network data

Data zones (DZs) are the key small area geographical units used in Scotland, with a total of 6,505 data zones (median population size =755) created from the 2001 Census [25] to reflect natural communities and composed of households with similar socioeconomic characteristics. In this analysis, only the 6,412 data zones from the mainland Scotland local authorities were considered as the transport network did not include the island local authorities of the Western Isles, Shetland and Orkney. The Scottish Executive six-fold Urban Rural Classification [26] was used to assign each data zone to one of six categories of urban/rural classification: Large Urban Areas Settlements (category 1), Other Urban Areas Settlements (category 2), Accessible Small Towns (category 3), Remote Small Towns (category 4), Accessible Rural Areas (category 5), and Remote Rural Areas (category 6). A representative list of all fixed recreational physical activity facilities in Scotland supplied by **sport**scotland, the national agency for sport [27], was mapped. Using the methods employed in our previous papers [19, 20] to take into account the effect of population density, we also adjusted the number of accessible facilities for the population of each DZ, as estimated from the 2001 Census, by calculating the mean number of accessible facilities per 1,000 individuals.

Information on the number of accessible physical activity (PA) facilities per 1,000 individuals in each data zone was derived by considering routes on foot, by bike, by car and by bus from the population weighted centroid of each data zone to each facility using Geographic Information System (GIS) software, imposing a maximum travel time threshold and dividing the number of accessible facilities by the population size of the data zone. Further information about the physical activity facilities included in the database (examples include sports centres, swimming pools and tennis courts) is documented elsewhere [28].

The transport network was created using TransCAD software version 5.0 [29] which combines GIS with transport planning functions. A transport network was created by importing data from the Ordnance Survey Integrated Transport Network layer covering mainland Scotland into TransCAD. Routes on which walking and cycling are not permitted, such as motorways, were removed from the network when deriving the number of accessible facilities available for these transport modes, with average speeds of 5 km/h [30] and 14 km/h [31] assumed for walking and cycling respectively. The car network was created to represent uncongested road conditions, with the speed limit of the road type adopted as the travel speed in the analysis and time penalties introduced for turning movements on the road [32]. The bus network was created using bus timetable information available from the National Public Transport Data Repository (https://data.gov.uk/dataset/nptdr) which contains details of bus stop locations and scheduled bus services in Scotland. The network reflected the bus services available during a weekday inter-peak period (between 10 am and 4 pm on a Wednesday) in October 2007. Bus stop waiting times were limited to five minutes, on the assumption that passengers would know the bus timetable and avoid excessive waiting times. Further information about the transport networks used in this analysis is documented elsewhere [20].

Routes were plotted from the population weighted centroid of each data zone to each of the physical activity facilities, assuming that travellers would take the shortest possible route by distance for walking or cycling and the fastest route by car or bus. Maximum travel times of 20 and 30 min were imposed to compute the number of facilities accessible from each data zone by each mode of transport.

### Statistical analysis

Univariable linear regression models were used to examine associations between each of the explanatory variables and BMI. Multilevel multivariable linear regression models were then used to examine associations between the number of accessible physical activity facilities per 1,000 individuals and BMI, adjusting for data zone and local authority. Separate models were estimated for each mode of transport (walking, cycling, car or bus), maximum travel time (20 min or 30 min) and facility ownership (all, public or private).

Where statistically significant associations were identified, the criteria described by Baron and Kenny [33] were adopted to determine whether or not physical activity participation mediated the relationship. The first criterion was that the explanatory variable of interest (accessibility) should be significantly associated with the putative mediator (participation). This was assessed using multilevel multivariable multinomial regression models. The second criterion was that the putative mediator (participation) should be significantly associated with the response variable (BMI) adjusting for other covariates of interest. The final criterion was that the inclusion of the putative mediator in the original model should reduce the association between the explanatory variable and the response variable, with the reduction in the magnitude of the regression coefficient being used to indicate the extent of the mediation.

The statistical analysis was carried out using MLwiN version 2.22 [34]. Analysis involving multiple multilevel regression models did not include adjustment for multiple comparisons. Although this is often argued to increase the possibility of chance findings, following Rothman’s argument [35] that adjustments for multiple comparisons are not necessary and can possibly lead to incorrect conclusions that there is no evidence of an association when there is, the unadjusted results are presented.

## Results

The sample included in the analysis comprised 2,860 men and 3,505 women with a mean age of 49.1 years, of whom 77 % had access to a car and 63 % lived in urban areas (Table 1). The mean BMI of the sample was 27.3 kg/m^2^ (Table 2). There was strong evidence of an association between age and BMI (*p* < 0.001) and there was also evidence that participants with no longstanding illness had a lower mean BMI than those with a limiting or non-limiting longstanding illness; that lower social class was associated with a higher mean BMI; and that participants with the worst quintile of dietary quality had a lower mean BMI than those in the middle quintile. There was no evidence of a difference in mean BMI between males and females, or between those with and without access to a car, but there was evidence of an association between BMI and the PA measures (data now added to Table 2).

**Table 1:** Sample characteristics

	Number	Percent
Individual level	6,365	
Gender		
Male	2,860	44.9
Female	3,505	55.1
Car availability		
Yes	4,887	76.8
No	1,478	23.2
Longstanding illness		
Limiting	1,705	26.8
Non-limiting	1,004	15.8
None	3,656	57.4
Social class		
Managerial	1,960	30.8
Intermediate	1,259	19.8
Routine	2,906	45.7
Other	240	3.8
DQI quintile		
1 (worst diet)	1,283	20.2
2	1,324	20.8
3 (middling)	1,146	18.0
4	1,368	21.5
5 (best diet)	1,244	19.5
Number of days of 20 mins + moderate/vigorous activity in 4 weeks		
None	1,466	23.0
1–11	2,349	36.9
12+	2,550	40.1
Occasions of sport activity of 15 mins + in 4 weeks		
None	3,535	55.5
1–11	1,655	26.0
12+	1,175	18.5
DZ level		
Urban/Rural		
1 (large urban)	2,063	32.4
2 (other urban)	1,951	30.7
3 (accessible small town)	592	9.3
4 (remote small town)	357	5.6
5 (accessible rural)	864	13.6
6 (remote rural)	538	8.5
	Mean (SD)	Min, Max
Age (years)	49.1 (17.2)	16.0, 95.0
Accessibility measure	Mean (SD)	Min, Max
Walking		
PA facilities (all) within 20 min	14.1 (12.6)	(0.0, 90.0)
PA facilities (all) within 30 min	29.3 (26.8)	(0.0, 176.1)
public PA facilities within 20 min	4.6 (4.9)	(0.0, 32.4)
public PA facilities within 30 min	9.5 (8.8)	(0.0, 68.6)
private PA facilities within 20 min	2.8 (3.6)	(0.0, 32.4)
private PA facilities within 30 min	5.9 (7.0)	(0.0, 62.2)
Cycling		
PA facilities (all) within 20 min	85.8 (88.3)	(0.0, 795.2)
PA facilities (all) within 30 min	166.9 (182.3)	(0.0, 1229.0)
public PA facilities within 20 min	26.4 (24.2)	(0.0, 162.7)
public PA facilities within 30 min	50.7 (48.9)	(0.0, 298.0)
private PA facilities within 20 min	17.4 (21.2)	(0.0, 208.4)
private PA facilities within 30 min	34.7 (42.8)	(0.0, 290.7)
Bus journey		
PA facilities (all) within 20 min	50.8 (69.1)	(0.0, 945.2)
PA facilities (all) within 30 min	204.3 (250.4)	(0.0, 2005.0)
public PA facilities within 20 min	15.8 (19.0)	(0.0, 232.2)
public PA facilities within 30 min	63.7 (69.8)	(0.0, 502.7)
private PA facilities within 20 min	10.2 (16.2)	(0.0, 248.6)
private PA facilities within 30 min	41.4 (54.5)	(0.0, 400.4)
Car journey		
PA facilities (all) within 20 min	1194.0 (1024.1)	(0.0, 4678.0)
PA facilities (all) within 30 min	2086.0 (1611.6)	(1.9, 7011.0)
public PA facilities within 20 min	394.1 (343.3)	(0.0, 1653.0)
public PA facilities within 30 min	702.9 (555.4)	(0.0, 2310.0)
private PA facilities within 20 min	240.2 (203.4)	(0.0, 891.2)
private PA facilities within 30 min	418.8 (315.3)	(0.0, 1335.0)

**Table 2:** BMI by individual explanatory variables

Variable	Mean BMI (95 % C.I.)	Minimum	Maximum	*p*-value*
Gender				0.87
Male	27.3 (27.1, 27.5)	15.7	47.4	
Female	27.3 (27.2, 27.5)	15.1	49.7	
Car availability				0.23
Yes	27.4 (27.2, 27.5)	15.6	49.0	
No	27.2 (26.9, 27.5)	15.1	49.7	
Limiting longstanding Illness				<0.001
Limiting	28.5 (28.3, 28.7)	15.1	49.7	
Non-limiting	28.1 (27.8, 28.4)	15.7	48.8	
None	26.6 (26.4, 26.7)	15.2	48.3	
Social class				<0.001
Managerial	27.1 (26.9, 27.4)	17.3	49.0	
Intermediate	27.6 (27.3, 27.8)	15.6	48.3	
Routine	27.5 (27.3, 27.7)	15.1	49.7	
Other	25.4 (24.8, 26.1)	15.7	46.7	
DQI quintile				<0.02
1 (worst diet)	26.9 (26.7, 27.2)	15.1	49.7	
2	27.5 (27.2, 27.7)	15.6	48.9	
3 (middling)	27.6 (27.3, 27.9)	16.4	46.0	
4	27.4 (27.1, 27.6)	15.2	45.6	
5 (best diet)	27.3 (27.0, 27.6)	16.4	48.4	
Number of days of 20 mins or more moderate/vigorous activity in 4 weeks				<0.001
None	28.14 (27.4,28.9)	15.07	49.67	
1–11	27.63 (27.0,28.3)	15.64	48.95	
12 or more	26.58 (26.0,27.2)	15.17	47.42	
Occasions of sport activity of 15 mins or more in 4 weeks				<0.001
None	27.81 (27.1, 28.5)	15.07	49.67	
1–11	26.89 (26.1, 27.6)	15.36	48.28	
12 or more	26.49 (25.6, 27.4)	15.90	47.42	
Overall	27.3 (27.2, 27.5)	15.1	49.7	-

*p-values from univariable linear regression models with response BMI

Associations between 24 separate accessibility measures and BMI were tested. There was evidence of an association between almost a third (7/24) of these measures and BMI after adjusting for age, gender, longstanding illness, car availability, social class, dietary quality and urban/rural classification in separate multilevel multivariable models, all in the expected (negative) direction indicating a reduction in mean BMI with increasing accessibility of facilities (Table 3). The seven significant measures of accessibility were those for all facilities within a 20 or 30 min walk or a 20 min cycle; and for private facilities within a 20 or 30 min walk, a 20 min cycle or a 20 min bus journey. There was no evidence of an association between BMI and any of the accessibility measures relating to bus travel to public facilities, or any of the measures of accessibility by car, in the multivariable models.

**Table 3:** Associations between accessibility of facilities and BMI

Accessibility measure (rate per 1000)	Coefficient (SE)	*p-*value	Adjusted^a^ coefficient (SE)	Adjusted *p*-value
Walking				
PA facilities (all) within 20 min	–0.021 (0.006)	<0.001	–0.015 (0.006)	**0.02**
PA facilities (all) within 30 min	–0.012 (0.003)	<0.001	–0.008 (0.003)	**0.01**
public PA facilities within 20 min	–0.020 (0.014)	0.17	–0.015 (0.015)	0.31
public PA facilities within 30 min	–0.017 (0.008)	0.04	–0.008 (0.009)	0.37
private PA facilities within 20 min	–0.067 (0.019)	<0.001	–0.046 (0.019)	**0.02**
private PA facilities within 30 min	–0.052 (0.010)	<0.001	–0.035 (0.011)	**0.001**
Cycling				
PA facilities (all) within 20 min	–0.004 (0.0008)	<0.001	–0.003 (0.001)	0.02
PA facilities (all) within 30 min	–0.002 (0.0004)	<0.001	–0.001 (0.001)	0.09
public PA facilities within 20 min	–0.011 (0.003)	<0.001	–0.004 (0.004)	0.37
public PA facilities within 30 min	–0.006 (0.001)	<0.001	–0.003 (0.003)	0.28
private PA facilities within 20 min	–0.017 (0.003)	<0.001	–0.012 (0.004)	0.004
private PA facilities within 30 min	–0.007 (0.002)	<0.001	–0.003 (0.002)	0.13
Bus journey				
PA facilities (all) within 20 min	–0.004 (0.001)	<0.001	–0.002 (0.001)	0.09
PA facilities (all) within 30 min	–0.001 (0.0003)	<0.001	–0.0004 (0.0003)	0.23
public PA facilities within 20 min	–0.011 (0.004)	0.003	–0.004 (0.003)	0.16
public PA facilities within 30 min	–0.003 (0.001)	0.002	–0.001 (0.001)	0.27
private PA facilities within 20 min	–0.016 (0.005)	<0.001	–0.008 (0.004)	0.03
private PA facilities within 30 min	–0.005 (0.001)	<0.001	–0.002 (0.002)	0.22
Car journey				
PA facilities (all) within 20 min	–0.0001 (0.0001)	0.19	0.00002 (0.00008)	0.76
PA facilities (all) within 30 min	–0.0001 (0.0001)	0.23	–0.00001 0.00005)	0.84
public PA facilities within 20 min	–0.0003 (0.0003)	0.26	0.00007 (0.0002)	0.75
public PA facilities within 30 min	–0.0002 (0.0002)	0.20	–0.00004 (0.0001)	0.76
private PA facilities within 20 min	–0.0006 (0.0004)	0.15	0.00001 (0.00041)	0.99
private PA facilities within 30 min	–0.0003 (0.0003)	0.20	–0.00009 (0.00025)	0.72

^a^Multilevel linear model (individuals nested within DZs within local authorities) adjusting for age, gender, limiting longstanding illness, car access, social class, DQI, urban/rural classification

*SE* standard error

To address the first criterion for mediation by participation in physical activity, each of these seven significant accessibility measures was considered as a predictor of physical activity participation. Although some significant associations were observed in univariable multinomial regression models, none of the seven accessibility measures remained significantly associated with participation in any physical activity or sport or exercise activity in multivariable models after adjustment for age, gender, social class, longstanding illness, dietary quality, car availability or urban/rural classification (data available on request). It therefore did not appear that the measures of physical activity participation in the Scottish Health Survey were capable of meeting the criteria to be identified as mediators of the relationship between accessibility of recreational physical activity facilities and BMI. Formal tests of the remaining Baron and Kenny criteria were therefore not performed.

## Discussion

In our Scotland-wide study, we found some evidence of an association between proximity to physical activity facilities and individual BMI, with almost a third of the accessibility measures we examined exhibiting a significant negative association with BMI. There were no significant associations between BMI and access to publicly owned physical activity facilities by any of the travel accessibility measures when we explored these separately by facility ownership. Although we found small effect sizes, these would nevertheless be important if adopted across a whole population [36]. We have previously reported that there are more public physical activity facilities in deprived areas across mainland Scotland [19] but this apparent advantage does not seem to be reflected in BMI levels, since obesity is more prevalent among people (particularly women) living in deprived areas [37]. It may be that other factors beyond transport access *per se* are more important; for example, public transport can be viewed as of inferior status or less comfortable [38, 39]. Some studies have found that residents’ perceptions of the pleasantness or safety of their neighbourhood influences their use of different types of local facilities that might promote physical activity [40]. The cost of using facilities [41] and their perceived availability may be more important than transport access which is objectively measured [42–44] and the extent to which different groups perceive physical activity facilities as suitable for their needs may be a contributory factor [45]. It may be that in designing interventions to encourage participation in physical activity, other factors such as facilities for childcare may be needed [46]. Psychosocial factors such as lack of confidence or anxieties over one’s appearance, or having someone to go with, have also been shown to be important [47, 48]. The role of transport access in relation to physical activity opportunities may be more important for some groups, e.g. the elderly [49–51], among whom a more tailored approach to promoting access may be required.

A strength of our study is that we developed a novel transport network across a whole country and were able to match this to data on the body size of individual participants in a large population survey. To our knowledge this has never been done before. A limitation of our study is that 18 % of the sample had missing or improbable values for BMI. Although there is increasing use of multiple imputation of missing data [52], as much of our missing data was in the outcome variable and we had no auxiliary variables in the survey data which we felt would predict missing objectively measured height and weight we chose to conduct a complete case analysis. Although complete case analysis assumes the data are Missing Completely At Random (MCAR), complete case analysis with covariate adjustment has been found to yield unbiased estimates of associations between exposures and outcomes when the data are Missing At Random (MAR) [53]. The Scottish Health Survey PA measure is limited in that it relies on self-report, but it would be challenging and costly to obtain objectively measured PA (e.g. through accelerometry) on over 8,000 respondents. Moreover, the self-report module covers a wide range of activities and a similar module has been validated against objectively measured PA [54]. The use of general rather than specific measures of PA such as walking and sports participation reflects the need for breadth (rather than depth) in general national population surveys. A further limitation of our study is that the Scottish Health Survey does not contain information on whether individuals actually used any of the facilities we examined. Therefore, we cannot assume that a given individual actually benefited from the variety or availability of facilities accessible from his or her local area, or that this explains the association with BMI. Other factors, unmeasured in our study, may also be relevant. Firstly, in relation to the physical environment, people may undertake physical activity in different settings. For example, some groups may prefer to exercise in open space [55] or may prefer to travel further afield to use particular facilities which are not in their area. Indeed, it may be more convenient for some people to use facilities near their place of work [56] rather than their immediate area of residence, whereas our analysis is predicated on respondents’ area of residence and we did not have information on where they worked. It is interesting to note here that in our study there was no association between BMI and access to facilities within 20 min by car, suggesting that a comparative lack of knowledge of the locations and characteristics of facilities further from respondents’ homes may be a contributory factor [55]. Secondly, we cannot discount the possibility that individuals may self-select into areas with a range of opportunities to be physically active [11]. However, this may be a more salient issue for those who have more choice over where they live, e.g. home owners rather than those who are bound by the availability and location of public sector housing (the predominant housing tenure in deprived areas in Scotland). Thirdly, social and cultural factors may contribute; for example, some studies have found that low income groups are more likely than higher income groups to perceive that there are fewer local facilities than actually exist on the ground [40, 49, 57]; in addition, social norms for health related behaviours may vary across different neighbourhoods and networks [58]. Fourthly, it may be that the food environment is more important for obesity than the physical activity environment [9]. However, we were unable to investigate the role of the local food environment on BMI as we know of no readily available single source dataset on the food environment across Scotland comparable to the **sport**scotland dataset. We have, however, controlled for dietary quality in our study. Finally, we lack information on any differences in accessibility of facilities by bus (or indeed by car) between 2003, when the physical activity data were collected, and 2007, when the facilities were ascertained. It is unlikely that there would have been significant changes in the locations of facilities, given that land use change tends to happen relatively slowly, and smaller local changes are likely to have balanced out across the country and in our analysis. Changes in the provision of public transport could also have taken place. The annual vehicle-kilometres (km) provided by local bus services in Scotland increased by 7.6 % between 2003/04 (369 million km) and 2007/08 (397 million km) [59]. However, this proportionate increase overestimates any change in accessibility as conceived in our analysis, which is based on the travel time between data zone centroids and sports facilities. More services (and more vehicle-kilometres) do not necessarily mean faster journey times (and hence increased accessibility) overall. In a deregulated public transport system, such data are more likely to reflect increases in service frequency on more popular routes, which would have only a marginal effect on our measures of accessibility.

## Conclusions

Our study has found some evidence that access to recreational facilities by different forms of transport is associated with obesity, and whilst we did not find this for all facilities, this may reflect the limitations of our data. Improving access to, and knowledge of, health-promoting facilities for those on low incomes may offer an important contribution to reducing the population prevalence of obesity.

## Abbreviations

BMI, body mass index; DQI, Dietary Quality Index; DZs, Data zones; GIS, Geographic Information System; Km, kilometres; NS-SEC, National Statistics Socio-economic Classification; PA, physical activity
